# Case Report: Hives and faints, an unusual affair

**DOI:** 10.12688/f1000research.141339.2

**Published:** 2024-07-09

**Authors:** Saket Toshniwal, Jiwan Kinkar, Sunil Kumar, Sourya Acharya, Madhukar Tikas, Isha Sahai, Benumadhab Ghosh, Suhit Naseri

**Affiliations:** 1 General medicine, Datta Meghe Institute of Higher Education and Research, Wardha, Maharashtra, 442001, India; 2 Neurology, Datta Meghe Institute of Higher Education and Research, Wardha, Maharashtra, 442001, India; 3 General medicine, Datta Meghe Institute of Higher Education and Research, Nagpur, Maharashtra, 442001, India; 4 Pathology, Datta Meghe Institute of Higher Education and Research, Wardha, Maharashtra, 442001, India

**Keywords:** Cholinergic urticaria, sweating, nonsedating antihistamics, syncope

## Abstract

Cholinergic urticaria (CholU) is a rare condition characterized by itchy hives in the form of 1-4 mm small, raised wheals on skin, which are short-lived for duration of 15 to 20 minutes; they are caused by stimuli associated with sweating such as from physical exercise. CholU is also known as cholinergic angioedema urticaria.

Hereby, we present a case report of a 42-year-old male with ChoIU who presented with systemic manifestation in the form of recurrent attacks of syncope. Diagnosis was made after a detailed history taking and all cardiac and neurological evaluations done that were normal, and widespread literature research was done to rule out other causes of syncope as systemic symptoms are rarely seen in ChoIU. His IgE antibodies levels were highly increased. He was managed with nonsedating antihistamines and health education regarding the rare condition.

## Introduction

Cholinergic urticaria (ChoIU) is one of the rare forms of physical urticaria. It is characterized by itchy, pinpoint size wheals which are generally triggered by heat, exercise, mental and physical stress, foul smell and consumption of spicy food. ChoIU is mostly a local reaction and systemic manifestations are rare, seen only in 10% of these cases. Following the exposure to the trigger factor, the onset of CholU occurs rapidly within a few minutes. The average duration of ChoIU symptoms is about 80 minutes, although they can continue up to one to two hours. ^1^^–^^3^

The rarely observed severe cases of CholU, a wheal-flare reaction, may be seen along with systemic involvement, characterized by symptoms like difficult breathing, wheezing, or abdominal pain. ^4^ Sweating associated with urticaria is the main diagnostic feature of CholU in addition to a type I allergic reaction. ^5^ The age group in which CholU is most commonly seen in 20 to 30 years, and there is no sex predominance reported among ChoIU patients.

The diagnosis is made based on the patient’s medical history and physical examination, although provocation tests are necessary to confirm CholU. The diagnosis of CholU has previously been made using strenuous exercise and passive warming to increase the body’s core temperature. ^2^ Another test involved injecting 100 g of methacholine intradermally with 0.1 mL of saline solution, which resulted in a positive CholU reaction within a minute. ^2^ Recently, two approaches have been proposed to examine patients with CholU suspicions: (i) Patients first exercise for 30 minutes on a treadmill or bicycle trainer, increasing their heart rate by 3 beats per minute; (ii) a passive warming test: a person stays in a 42 degrees Celsius (°C) bath. Following an increase in body temperature of 1°C above the baseline, patients continue the passive warming test for an additional 15 minutes. The presence of tiny wheals during the test and for 10 minutes after it stops indicates a positive result. ^6^

We share our case to raise awareness of the disorder, shed additional light on the diagnosis, and minimise unnecessary costly studies and delays in diagnosis due to the rarity of systemic involvement seen in CholU.

## Case presentation

A 42-year-old Asian man labourer by occupation who had previously fainted after physical activity visited the emergency room in our hospital. Before the incidence of fainting, a rash had been previously noticed. The patient also provided a history of syncopal attacks twice after arguments with relatives which begun with severe itch, salivation and blurry vision. These symptoms were also associated with frequent episodes of generalized discomfort, nausea and vomiting.

**Figure 1.**
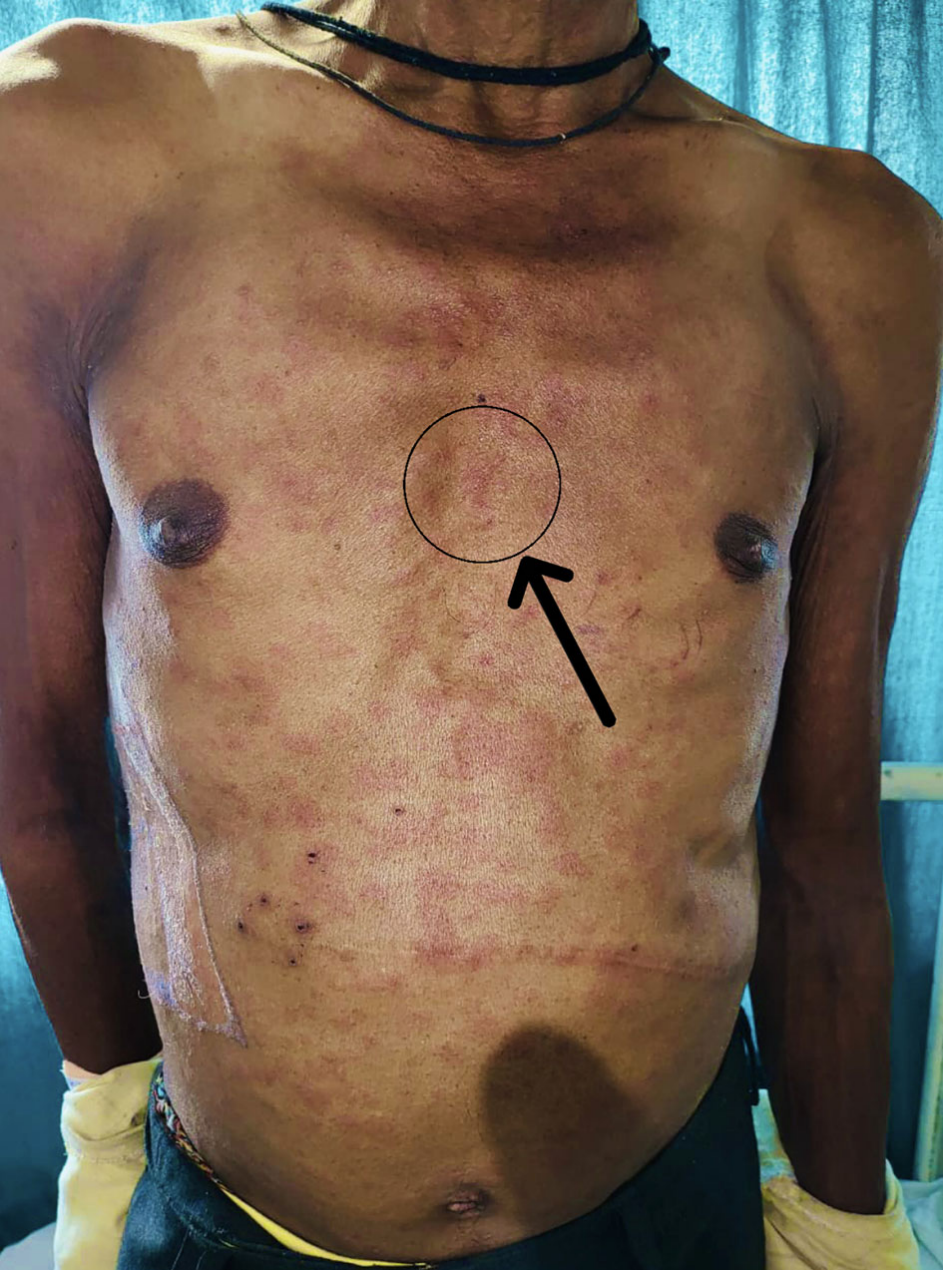
Hypersensitivity reaction (papular lesions) on the patient’s body with black arrow.

**Figure 2.**
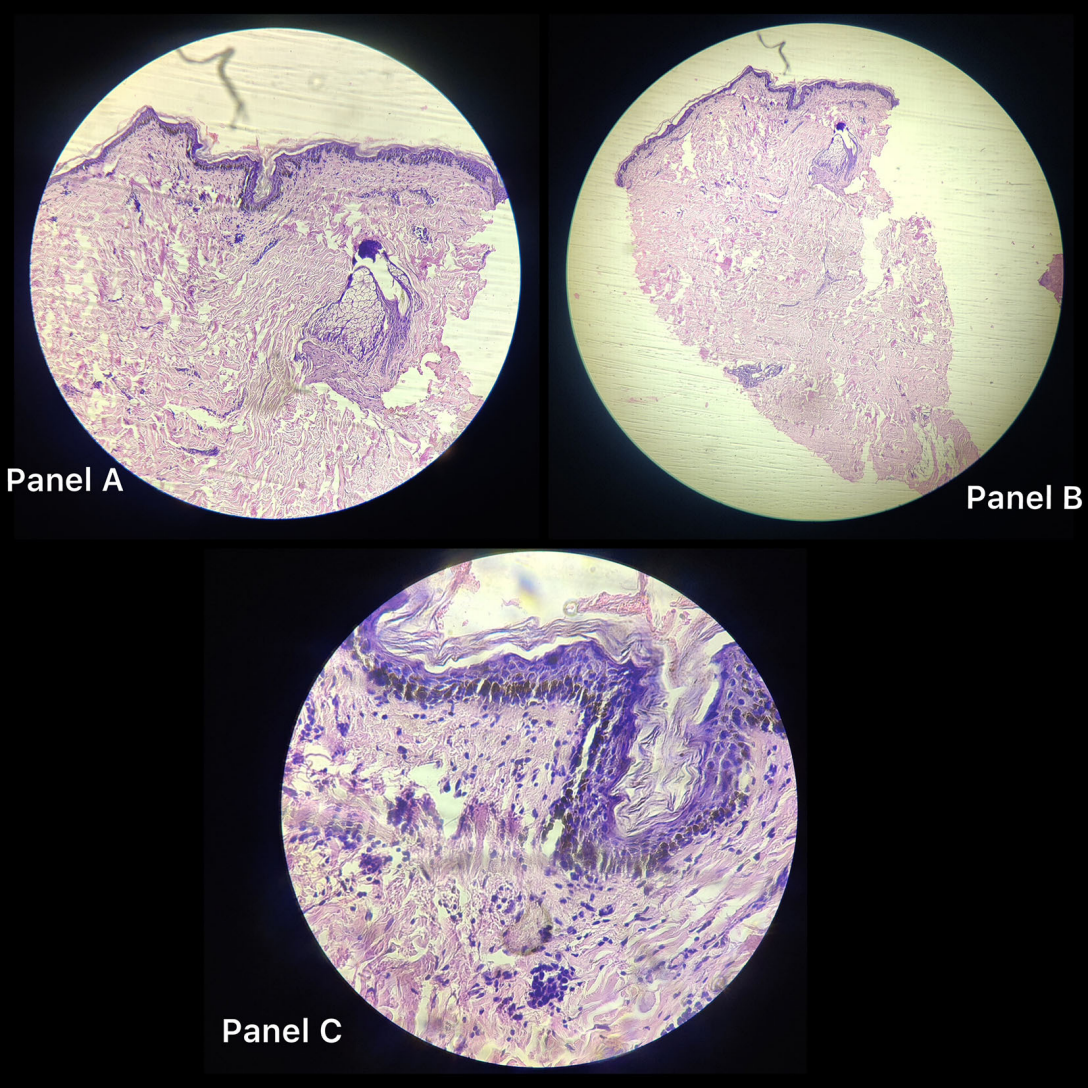
Panel A and B: Histopathological images of skin biopsy from anterior chest wall showing thinned out and atrophied lining epithelium. Panel C: Deeper tissue show increased collagenosis, loss of adnexal structures and scattered chronic lymphocytic inflammatory infiltrate in superficial dermis on histopathology.

Further investigation revealed that the patient had previously had papular lesions ( Figure 1), itching, and burning after being exposed to various stimuli, including sunshine, hot meals, unpleasant odours, emotions, and exercise, as well as comparable bouts of fainting. The bout of fainting was followed by an unplanned recovery. The wheals and rash also disappeared. There was no history of similar occurrences in either the family or the individual, and there was no family history of any other allergies. After the patient was examined, it was discovered that his cardiac profile, comprising an electrocardiogram, a 2D echocardiogram, a stress test, and holter monitoring, were all within normal limits. Similar to this, the neurological profile, which involved testing including brain MRIs, electroencephalograms, and biochemical analyses, was completed and confirmed to be normal. Provocation tests were done via heavy exercise and passive warming to elicit an allergic response, and were found to be positive for cholinergic urticaria. A section from skin biopsy from anterior chest wall ( Figure 2) showed thinned-out and atrophied lining epithelium (panel A and B). Deeper tissue showed increased collagenosis, loss of adnexal structures and scattered chronic lymphocytic inflammatory infiltrate in superficial dermis on histopathology as shown in Figure 2, panel C. Polymorphic light eruption which is seasonal (generally occurs in spring) and has symmetrically distributed polymorphic skin lesions was also ruled out; maculopapular cutaneous mastocytosis was also ruled out as the lesions did not urticate within a few minutes when rubbed; similarly, Sweets Syndrome was also ruled out as the rash did not have a pseudo vesicular aspect. Other differential diagnoses like mast cell activation syndrome, erythema annulare centrifugum, bullous pemphigoid, angioedema, amonf others, were also ruled out based on specific signs and symptoms of each. When all the other causes of syncope were ruled out, serum IgE measure was done which was grossly elevated to 1800 IU/L (Normal range 1.31–165.5 IU/L).

Based on the above history, clinical picture, and investigations, after a thorough literature search, a diagnosis of CholU with cardiovascular involvement as syncope and allergic vasodilation or hyperventilation with stress and exercise was made. The patient was treated with oral fexofenadine 120mg bd, to which he responded, and as of follow-up after six months, he was symptom-free on therapy.

## Discussion

CholU is a rare form of inducible urticaria, and was reported by Duke in 1924 for the first time. ^3^^,^^5^ In the present case the patient reported with highly pruritic pinpoint wheals associated with the very infrequent systemic symptom of fainting during physical exercise. According to previous reports CholU is generally seen in the age group of 20 to 28 years ^2^; however, in the present case the patient was 42 years old. Based on a previous hypothesis, it is said that patients with CholU are usually hypersensitive to their own stress which results in the development of wheals. According to a theory that has been put forth, patients typically become hypersensitive to unidentified chemicals in their perspiration and develop skin wheals. ^6^^,^^7^ The patient in the current report experienced a similar response. The pathogenesis of CholU has been attributed to disturbed AChE. A number of systemic manifestations of CholU have been documented, including dysphagia, dysphonia, inspiratory stridor, lower airway symptoms like wheezing, coughing, chest tightness, and dyspnea, gastrointestinal involvements like generalised discomfort, nausea, vomiting, abdominal pain, and diarrhea, and cardiovascular manifestations like presyncope, syncope, and palpitations. ^6^^–^^9^ A study by Vadas *et al.* on 19 cases of ChoIU with cardiovascular system involvement having syncopal attack revealed mast cell degranulation as acetylcholine levels rise. ^4^ According to a Davis *et al*. study, local pruritus and urticaria are caused by degranulation from skin mast cells, whereas systemic symptoms are thought to be caused by histamine released from circulating basophils. ^10^

In this case, the exact mechanism of fainting and its association with CholU cannot be postulated. Because systemic involvement in this disorder is rather uncommon, there is frequently a lag between the beginning of symptoms and diagnosis. Additionally, treatment for a normally treatable ailment is delayed and other pointless studies are recommended in between. Non-sedating antihistamines are the mainstay of treatment for CholU, while ketotifen, montelukast, propranolol, and danazol have been suggested in cases where the antihistamines are ineffective.

Non-sedating antihistaminics (nsAH1) are recommended as the first line of treatment for ChoIU and trigger factor escape, under the most recent guidelines of EAACI/GA2LEN/EDF/UNEV ^9^ consensus recommendations for CholU management.

## Conclusions

A detailed history and thorough knowledge about CholU will prove beneficial in early diagnosis of this rare condition. This will ultimately help in saving time and money behind unnecessary investigations, also, mental trauma associated with the diagnostic dilemma to the patient. Effective education about CholU and allergic vasodilation and hyperventilation in presence of mental and physical stressors will aid in understanding the triggers and avoiding them; and thus will help in successful management of this unusual and rare condition.

Also, physicians should be well aware of this rare condition in individuals, especially in young adults, who present with recurrent syncopal attacks and should consider cholinergic urticaria as a possible differential diagnosis in presence of various triggers precipitating syncopal attacks along with rash over the body.

### Patient consent

Written informed consent for publication of their clinical details and clinical images was obtained from the patient.

## Data Availability

All data underlying the results are available as part of the article and no additional source data are required.
