# Supplementary material for: Case Report: Hives and faints, an unusual affair
Source: F1000Res. 2024 Jul 9;12:1193. Originally published 2023 Sep 25. [Version 2] doi: 10.12688/f1000research.141339.2 (PMC11258541; doi:10.12688/f1000research.141339.2)
Supplement: Peer review history

# Reviewer report: Reviewer response for version 2

**DOI:** 10.5256/f1000research.168833.r300984

**Published:** 2024-07-18

**Authors:** Thamir A Kubaisi

**Affiliations:** 1 Department of Dermatology, University of Anbar, Ramadi, Al Anbar Governorate, Iraq

**Dear all the Editorial Team, F1000Research**

**I approve the well-done changes and corrections.**

Are enough details provided of any physical examination and diagnostic tests, treatment given and outcomes?

Yes

Is the case presented with sufficient detail to be useful for other practitioners?

Yes

Is sufficient discussion included of the importance of the findings and their relevance to future understanding of disease processes, diagnosis or treatment?

Yes

Is the background of the case’s history and progression described in sufficient detail?

Partly

Reviewer Expertise:

NA

I confirm that I have read this submission and believe that I have an appropriate level of expertise to confirm that it is of an acceptable scientific standard.

# Reviewer report: Reviewer response for version 1

**DOI:** 10.5256/f1000research.154774.r284821

**Published:** 2024-06-02

**Authors:** Thamir A Kubaisi

**Affiliations:** 1 Department of Dermatology, University of Anbar, Ramadi, Al Anbar Governorate, Iraq

-Cholinergic urticaria (CholU) is not a rare problem comprising about 30% of physical urticaria. However the associated syncopal attack is considered a sporadic condition

 -The signs and symptoms of CholU were aggravated by heavy exercise using a bicycle treadmill or stair-stepping within 15 minutes

 -According to the modified urticaria activity score, twice-daily form for CholU. The severity depends on

1. Duration of skin lesions at attach
2. Number of hives at each check (or number of palms covers pinpoint hives)
3. Itch, tingling intensity

-The accompanying symptoms such as generalized discomfort, acquired hypo hidrosis, nausea, vomiting and headache are missed in this case report.

 -Please read this paper carefully: *Kubaisi, TA. "Regular sports exercises as a supportive therapeutic choice in cholinergic urticaria." Journal of Pakistan Association of Dermatologists 33.4 (2023): 1298-1305.* *‏*

 -Ethical consideration was absent.

 -There are no clear objectives.: my advice is: the syncopal attach in a young person could be considered as rare presentation of CholU that may be missed by physician.

Are enough details provided of any physical examination and diagnostic tests, treatment given and outcomes?

Yes

Is the case presented with sufficient detail to be useful for other practitioners?

Yes

Is sufficient discussion included of the importance of the findings and their relevance to future understanding of disease processes, diagnosis or treatment?

Yes

Is the background of the case’s history and progression described in sufficient detail?

Partly

Reviewer Expertise:

Dermatology and venereology

I confirm that I have read this submission and believe that I have an appropriate level of expertise to confirm that it is of an acceptable scientific standard, however I have significant reservations, as outlined above.

## References

[1] : Regular sweating activities for the treatment of cholinergic urticaria with or without acquired idiopathic generalized anhidrosis. *Dermatol Ther* .2020;33(4) : 10.1111/dth.13647 e13647 10.1111/dth.13647 32445244

# Response

**Published:** 2024-07-09

**Authors:** Saket Toshniwal

**Affiliations:** General medicine, datta meghe institute of higher education and research, wardha, Wardha, Maharashtra, India

Respected Reviewer,

 Thank you for reviewing the article and providing us with your expert opinion.

 The article has been refined and rectified as per your comments.

 Thank you.

# Reviewer report: Reviewer response for version 1

**DOI:** 10.5256/f1000research.154774.r270954

**Published:** 2024-05-17

**Authors:** Ocílio Ribeiro Gonçalves

**Affiliations:** 1 Federal University of Piauí (UFPI), Teresina, Brazil

.Minor points: This is a very interesting case report with a good management description. However, the authors could explain better what is the relationship between vasovagal syncope, acetylcholine, histamine and mast cell and basophils in the discussion. The pathophysiology of Cholinergic urticaria (ChoIU) is essential to understand the clinical presentations of the patient.

 Major points: In discussion it is also necessary that the authors explain why the treatment with an antihistamine (like fexofenadine) could turn the patient symptom-free, based on the pathophysiology of the disease. A simple antihistamine could add quality of life to the patient treating an important cardiovascular symptom presentation.

Are enough details provided of any physical examination and diagnostic tests, treatment given and outcomes?

Yes

Is the case presented with sufficient detail to be useful for other practitioners?

Partly

Is sufficient discussion included of the importance of the findings and their relevance to future understanding of disease processes, diagnosis or treatment?

Partly

Is the background of the case’s history and progression described in sufficient detail?

Yes

Reviewer Expertise:

Dermatology, Neurology and Cardiology

I confirm that I have read this submission and believe that I have an appropriate level of expertise to confirm that it is of an acceptable scientific standard.
